# Characterization of a Novel Linezolid Resistance Gene *optrA* and Bacitracin Resistance Locus-Carrying Multiple Antibiotic Resistant Integrative and Conjugative Element ICE*Ssu*1112S in *Streptococccus Suis*

**DOI:** 10.1128/spectrum.01963-21

**Published:** 2022-02-16

**Authors:** Yingying Yang, Xiuhua Kuang, Rong-jia Han, Ya-jun Zhai, Dan-dan He, Jin-feng Zhao, Jian-hua Liu, Gong-zheng Hu

**Affiliations:** a College of Veterinary Medicine, Henan Agricultural University, Zhengzhou, China; b Henan University of Animal Husbandry and Economy, Zhengzhou, China; University of Nebraska-Lincoln

**Keywords:** *Streptococcus suis*, integrative and conjugative element, linezolid, bacitracin

## Abstract

Streptococcus suis strain 1112S was isolated from a diseased pig in a feedlot from Henan, China, in 2019. The isolate harbored a linezolid resistance gene *optrA*. WGS data revealed that the *optrA* gene was associated with a single copy ETAf IS*S1S*, in tandem with *erm*(B) and *tet*(O), located in a novel 72,587 bp integrative and conjugative element (ICE). Notably, this novel element, designated ICE*Ssu*1112S, also carried a novel bacitracin resistance locus. ICE*Ssu*1112S could be excised from chromosome and transferred to the recipient strain S. suis P1/7 with a frequency of 5.9 × 10^−6^ transconjugants per donor cell. This study provided the first description of the coexistence of *optrA* and a novel bacitracin locus on a multiple antibiotic resistant ICE and highlighted that ICE were major vehicle and contribute to the potential transfer of clinically relevant antibiotic resistance genes.

**IMPORTANCE** Antimicrobial resistance (AMR) caused by the imprudent use of antimicrobials has become a global problem, which poses a serious threat to treatment of S. suis infection in pigs and humans. Importantly, AMR genes can horizontally spread among commensal organisms and pathogenic microbiota, thereby accelerating the dissemination of AMR determinants. These transfers are mainly mediated by mobile genetic elements, including ICEs. In S. suis, ICEs are the major vehicles that contribute to the natural transfers of AMR genes among different bacterial pathogens. However, ICEs that carry *optrA* and bacitracin resistance locus are rarely investigated in S. suis isolates. Here, we investigated a S. suis isolate carrying an *optrA* and a novel bacitracin resistance locus, which were co-located on a novel multiple antibiotic resistant ICE*Ssu*1112S. Our study suggests that more research is needed to access the real significance of ICEs that horizontally spread clinical important resistance genes.

## INTRODUCTION

Streptococcus suis is one of the major pathogens of swine, which causes swine arthritis, septicemia, meningitis, endocarditis, abortions, and sudden death (1). Furthermore, S. suis is a zoonotic human pathogen affecting those in close contact with infected pigs or pig products (2). With the extensive use of antibiotics, the emergence of drug-resistant and especially multidrug-resistant (MDR) isolates have been frequently reported in recent years in S. suis (3). Horizontal gene transfer (HGT) remains a crucial driving force in bacterial evolution, especially in dissemination of antimicrobial resistance (AMR). Mechanisms of horizontal gene transfer are mainly mediated by mobile genetic elements (MGEs) and natural competence for DNA transformation in S. suis (2). The MGEs include integrative and conjugative elements, plasmids, insertion sequences, transposons, prophages, integrons and genomic islands (4). ICEs are major vehicles of lateral gene transfer, which can integrate into chromosomes, plasmids and other mobile elements in major streptococcal pathogens carrying resistance genes, even virulence genes (5, 6).

Oxazolidinones (such as linezolid and tedizolid), approved exclusively for use in human medicine (7), can effectively treat infections caused by methicillin-resistant Staphylococcus aureus, vancomycin-resistant *Enterococci*, penicillin-resistant Streptococcus pneumoniae and multidrug-resistant Mycobacterium tuberculosis (8). Oxazolidinones are also recognized as the last-resort antimicrobial agents for the control of clinical infections caused by MDR Gram-positive pathogens (9). Oxazolidinone resistance in Gram-positive and Gram-negative is mainly mediated by transferable resistance genes such as *optrA*, *cfr*, *cfr*(B), *cfr*(C), *cfr*(D), *cfr*(E) and *poxtA*, as well as mutations in 23S rRNA and ribosomal proteins (L3, L4, L22) (10–14). It is worth mentioning that *optrA* gene in the ΦSsuD.1-like prophages, genomic islands and putative ICEs in S. suis has already been found recently (14, 15). In *Streptococci* and *Enterococci*, IS*1216*-mediated recombination plays an important role in the dissemination of *optrA* (15, 16). However, there are only limited reports of emergence of *optrA* gene through other transfer mechanisms in S. suis.

Bacitracin is an antimicrobial that comprises a mixture of high-molecular-weight polypeptides produced by the organism Bacillus licheniformis. Bacitracin is used widely for topical applications in human medicine and has been suggested for oral use in the control of vancomycin-resistant *Enterococci* (17, 18). Recent studies characterized an acquired bacitracin resistance locus *bcrABDR* encoding high-level bacitracin resistance in Gram-positive pathogens (19). The Bce system comprising a two-component system (TCS) BceSR and membrane transporter BceAB to export bacitracin has been identified in Streptococcus mutans, Bacillus subtilis and E. faecalis (17, 20, 21). In S. suis of porcine origin, Huang et al. (18) reported first the bacitracin resistance locus *bcrABDR* that was flanked by two copies of IS*1216* elements and *optrA* gene, which co-located on an antibiotic-resistance-associated genomic island.

In this study, we identified a novel multiple antibiotic resistant ICE*Ssu*1112S in a S. suis strain that was isolated during a routine survey of lincomycin-resistant S. suis in diseased pigs in a feedlot from Henan, China in 2019. In addition to harboring *lnu*(C), for lincosamide resistance, ICE*Ssu*1112S carried a novel bacitracin resistance locus and *optrA* for linezolid resistance.

## RESULTS AND DISCUSSION

### Characteristics of isolate 1112S and its resistance mechanisms.

The isolated S. suis strain 1112S exhibited a intermediate MIC of linezolid (4 mg/liter). The PCR results showed that the isolate 1112S carried the *optrA* gene but not *cfr*, *cfr*(B), *cfr*(C) and *poxtA*. Also, it was a MDR strain, which was resistant to lincomycin, florfenicol, doxycycline, azithromycin and tylosin, but susceptible to tigecycline and rifampicin (Table 1). Unexpectedly, it also exhibited a high MIC of an antimicrobial bacitracin (>512 mg/liter). As expected, it was nonserotypeable and belonged to a new sequence type ST1615.

**TABLE 1:** MICs for S. suis 1112S, S. suis P1/7 and transconjugant T-1112S^*b*^

Isolate	MIC (mg/liter) for:								
	LZD^*a*^	FFC	LIN	DOX	TGC	AZM	TYL	RIF	BAC
S. suis 1112S	4	128	>512	16	<0.5	>512	>512	<0.5	>512
S. suis P1/7	<0.5	0.5	<0.5	<0.5	<0.5	<0.5	<0.5	512	<0.5
Transconjugant T-1112S	4	128	>512	16	<0.5	>512	>512	512	128

a LZD, linezolid; FFC, florfenicol; LIN, lincomycin; DOX, doxycycline; TGC, tigecycline; AZM, azithromycin; TYL, tylosin; RIF, rifampicin; BAC, bacitracin.

b S. suis 1112S was used as donor isolate while S. suis P1/7 was used as recipient isolate. T-1112S was transconjugant.

The chromosome of S. suis 1112S was 2,440,827 bp long with a G/C content of 41.42%. BLAST search for the acquired AMR genes revealed the presence of 7 antimicrobial resistance genes, including tetracycline resistance gene *tet*(O), aminoglycoside resistance gene (*ant[6]-Ia, ant[9]*), spectinomycin resistance gene *spw*, macrolide-lincosamide-streptogramin B resistance gene *erm*(B), phenicol and oxazolidinone resistance gene *optrA* and lincosamide resistance gene *lnu*(C) in 1112S. Interestingly, the *lnu*(C) gene was part of the MTnSag1 transposon previously described (22), but the gene was found in S. suis for the first time. Notably, WGS analysis showed that three resistance genes (*erm[B], optrA and tet[O]*) and a bacitracin locus were located on a novel integrative conjugative element, hereby named ICE*Ssu*1112S.

### Characterization of ICE*Ssu*1112S.

**FIG 1:**
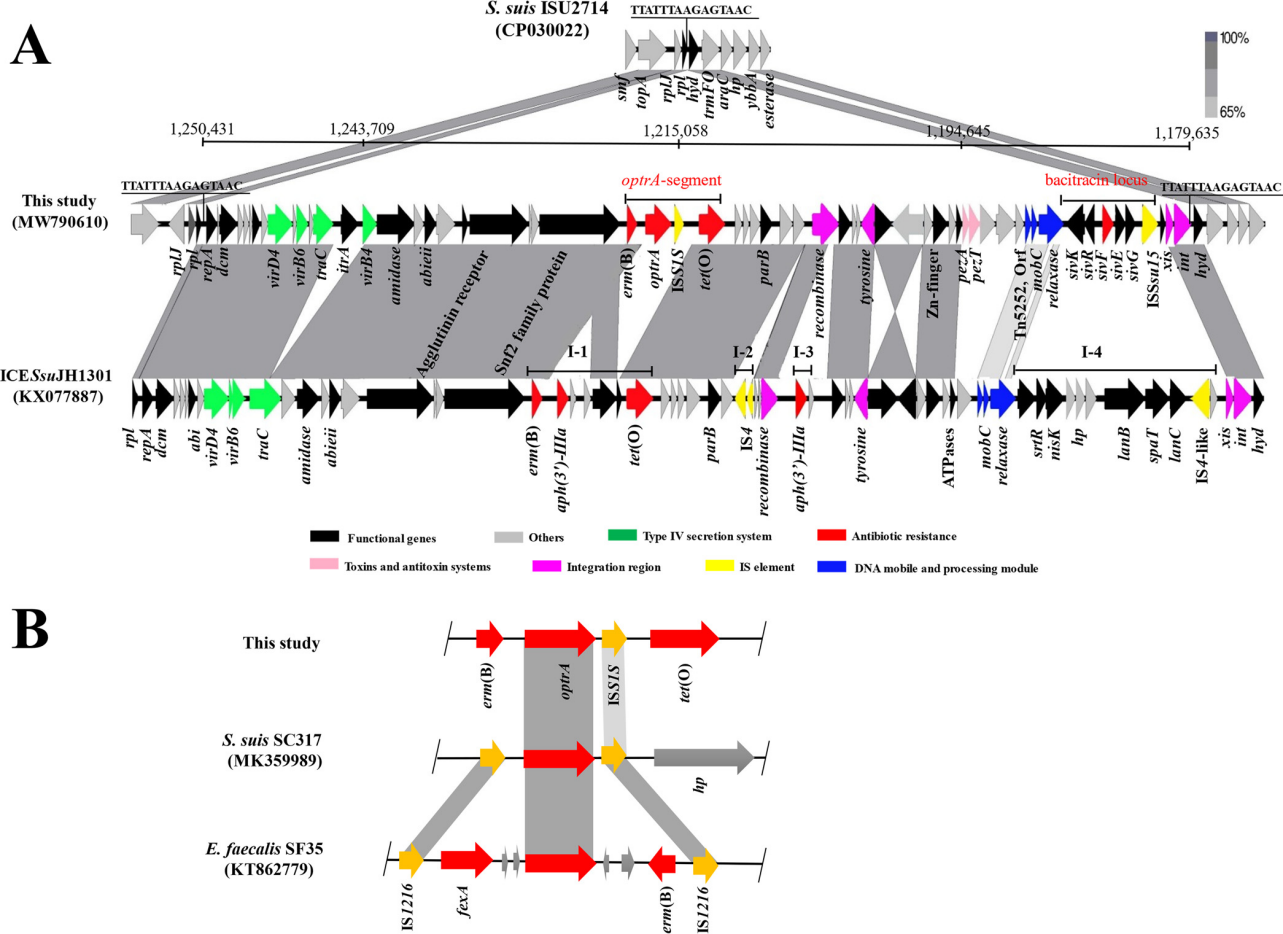
(A) Linear comparison of the ICE*Ssu*1112S with the homologous region of the genome of S. suis ISU2714 and S. suis ICE*Ssu*JH1301. There are other exogenous DNA insertion sites (I), namely, I-1, I-2, I-3, and I-4 in S. suis ICE*Ssu*JH1301. (B) Linear comparison of the *optrA*-containing segment in 1112S with that in S. suis strain SC317 (MK359989) and E. faecalis strain SF35 (KT862779). Genes and open reading frames are indicated as arrows, and their orientations of transcription are indicated by the arrowheads.

ICE*Ssu*1112S (72,587 bp), with average G/C content of 38%, was located between *rplL* and *hyd*, which encode a predicted 50S ribosomal protein L7/L12 and hydrolase, respectively (Fig. 1A). A 15 bp direct repeat (5′-TTATTTAAGAGTAAC-3′) was detected at both the left (L) and right (R) ends of ICE*Ssu*1112S, corresponding to the recognition site of the *att* integrase from the ICE*Sa*2603 family (Int_ICE_*_Sa_*_2603_) (23). The *rplL* locus is a known as insertion hot spot of mobile genetic elements, including ICEs in S. suis (4). Comparative sequence analysis showed that ICE*Ssu*1112S shared the most identity with S. suis ICE*Ssu*JH1301 (KX077877), which was found recently in China (73% coverage with 96% identity). Like numerous ICE*Sa*2603 family ICEs, ICE*Ssu*1112S consisted of a highly conserved genetic backbone involved in various functions, which comprised multiple types of genetic material consisting of resistance gene cluster, PezAT toxin-antitoxin system, a type IV secretion system (T4SS), and an integrase and excisase system (Fig. 1A). The conjugative transfer module (green boxes) was associated mainly with the type IV secretion system (T4SS) and included type-IV secretion system-like protein TraC. A series of complex proteins (VirD4, VirB6 and VirB4) were likely involved in the type IV secretion system, recently described for *Streptococci* (24), participating in conjugal transfer of the ICE with other members of the conjugation machinery, such as the relaxase (25). Immediately downstream of the type IV secretion system cluster is an ORF encoding amidase, which is an important hydrolytic enzyme in detoxification metabolism (26). Downstream, three ORFs coding for an agglutinin receptor, a calcium-binding protein, and an SNF2 family protein were found, respectively. The putative surface-associated agglutinin receptor can contribute to S. suis virulence by modulating the ability of the pathogen to adhere to host cells, the mechanism of which was previously reported in S. mutans (27). A predicted PezAT toxin/antitoxin system was mapped in the ICE*Ssu*1112S (pink boxes). Three predicted proteins (relaxase, MobC, and Tn5252 ORF10) may participate in the DNA mobility and processing module (blue boxes). A relaxase was required for bacterial conjugation, which nicked an origin of transfer (*ori*T) on the conjugative element and initiated the 5′-to-3′ transfer of one strand of the element into recipient cells (28). The integration and excision modules in ICE*Ssu*1112S were identified next to the *attL* site, and similar functional recombinase were mapped (purple boxes). This integrase corresponds to a tyrosine family site-specific recombinase related to integrase characterizing the ICE*Sa*2603 family. Importantly, the genetic structures of I-1 and I-4 in ICE*Ssu*1112S were different from the closely related ICE*Ssu*JH1301. These regions possess the *optrA*-containing segment and a bacitracin locus (corresponding to bases 38088 to 40902 and 3370 to 7393 in accession number MW790610, respectively), respectively (Fig. 1A).

### Genetic environment of *optrA* on ICE*Ssu*1112S.

The *optrA*-containing segment harbored a novel genetic structure. Downstream of the *optrA* gene was present a single copy of IS*S1S*, in tandem with *erm*(B) and *tet*(O) directly. Sequence analysis showed that the genetic environment of *optrA* differed distinctly from those in S. suis strain SC317 and E. faecalis strain SF35 (Fig. 1B). The *optrA* gene is usually flanked by two copies of IS*1216* elements (16, 18, 29), but we first found that *optrA* gene was flanked by a single copy of IS*S1S.* However, no circular intermediate carrying *optrA* was detected in the study, probably due to the lack of an IS*S1S* copy near one end of *optrA* gene. The *optrA*-containing segment was likely assembled with different parts from other ICEs or MGEs harboring resistance genes, involving a series of insertion and deletion events.

### Functional characteristics of bacitracin locus located in ICE*Ssu*1112S.

The bacitracin locus in this study comprised five ORFs, which showed high homology (93.6%) to a lantibiotic gene locus (*sivK*, *sivR*, *sivF*, *sivE* and *sivG*) in Streptococcus salivarius (accession number GQ857551). Homology comparison found that this bacitracin locus showed a low sequence identity to S. suis SFJ44, where the *bcrABDR* locus was located in the genomic island. Furthermore, these five ORFs were named *sivK*, *sivR*, *sivF*, *sivE* and *sivG*, respectively. *SivK* and *sivR* encoded two-component transcriptional response regulator and histidine kinase with 46% and 93% similarity to *BceSR* of S. mutans, respectively (20). *SivF* encoded ATP-binding cassette domain-containing protein (302 amino acids) with 41% protein identity and 98% similarity to *bcrA* of S. suis (305 amino acids) (accession number CP031970). These two ORFs (*sivE* and *sivG*) were located immediately downstream from *sivF* and encoded ABC transporter permease of 251 amino acids and 244 amino acids, respectively. The bacitracin locus was previously reported as a lantibiotic locus, which induced lantibiotic production and was present only in *S. salivarius* (30).

To further understand the characteristics of the bacitracin locus, the intact bacitracin locus was cloned into the shuttle vector pAM401. Electroporation of recombinant pAM401 into E. faecalis JH2-2 resulted in a bacitracin MIC of 128 mg/liter (pAM401-BAC). The result showed a 4-fold increase in the MICs of bacitracin, compared with JH2-2 or JH2-2 harboring empty vector (Table 2). These results demonstrate that the bacitracin locus comprised of a two-component system and ABC transporter (31), likely mediates bacitracin resistance. Interestingly, the bacitracin MIC for parental strain 1112S was >512 mg/liter, suggesting that other mechanisms might contribute to bacitracin resistance. In this study, a novel bacitracin locus in ICE*Ssu*1112S of S. suis was described for the first time.

**TABLE 2:** MICs of S. suis 1112S, E. faecalis JH2-2 and strains harboring recombinant vector or the corresponding empty vector

Bacterial strains	MIC (mg/liter) for: BAC
E. faecalis JH2-2	32
E. faecalis JH2-2+pAM401	32
E. faecalis JH2-2+pAM401-BAC	128
*S.suis* 1112S	>512

### Mobilization of ICE*Ssu*1112S.

**FIG 2:**
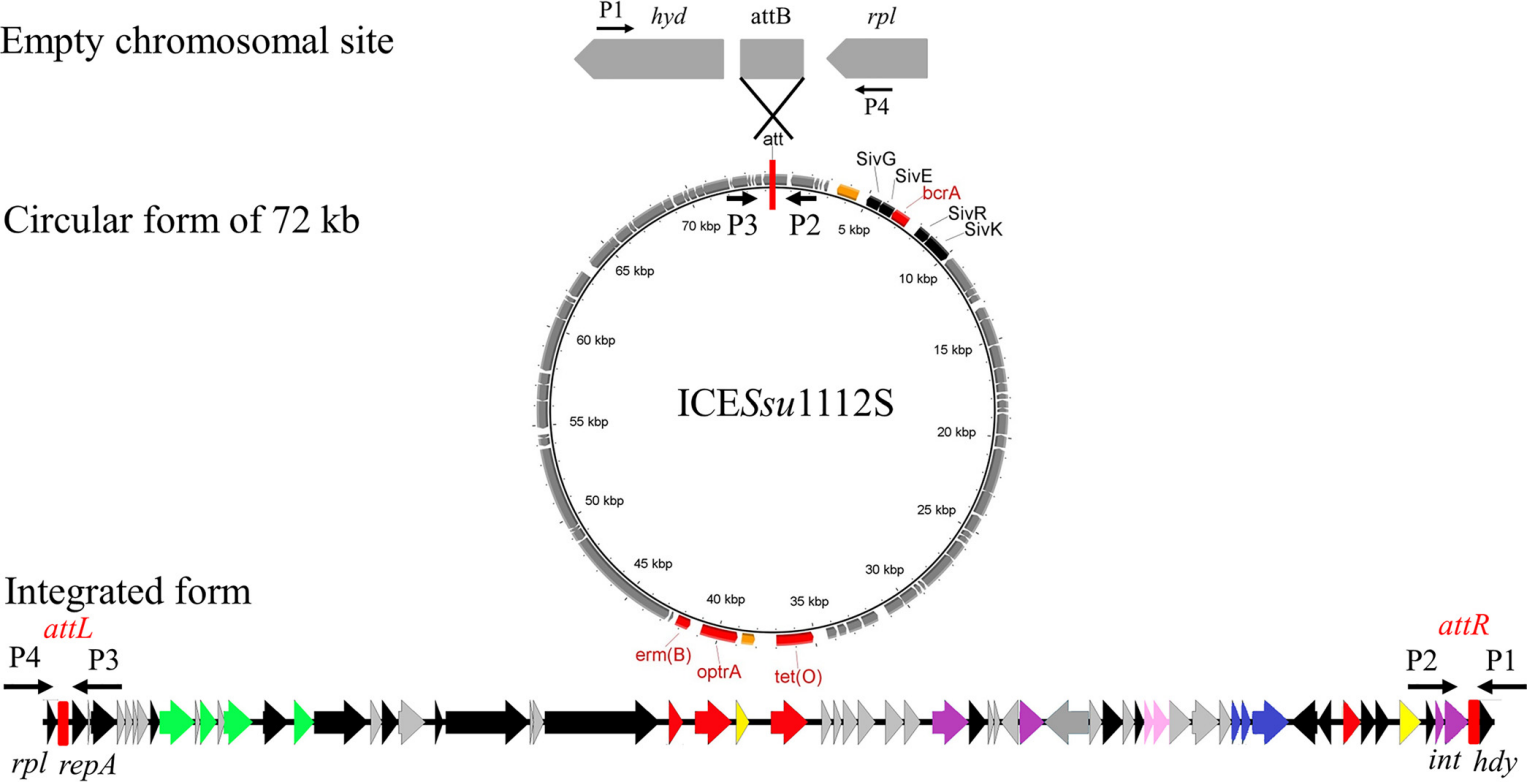
Schematic diagram of the integration and excision forms of ICE*Ssu*1112S in the chromosome of S. suis 1112S. The left and right red rectangles represent junctions (*attL* and *attR*), which can be formed by recombination. The direction and location of primers used to check the integration and excision forms of 72,587 bp are indicated by black arrows.

Due to the integrase gene recognizing the *att* site at the 3′ end of *rplL*, ICE*Ssu*1112S could be integrated into the bacterial chromosome. Excision enzymes can catalyze ICE excision from the bacterial chromosome leading to a circular extrachromosomal form of the ICE, which can be transferred by conjugation (Fig. 2) (32, 33). The integrated form and extrachromosomal circular form of the ICE*Ssu*1112S element were explored by PCR using primers P1-P4. If ICE*Ssu*1112S was present in the S. suis 1112S genome, primer pairs P1/P2 and P3/P4 would yield positive PCR products, whereas the primer pair P1/P4 would not. When ICE*Ssu*1112S generated the circular form by excising from the donor chromosome, PCR products would be obtained using primer P2/P3. S. suis strains 1112S and P1/7, as well as ddH_2_O, were used as the templates and the corresponding PCR products were shown in Fig. S1. When strain 1112S was cultured to logarithmic phase, four PCR products were detected simultaneously using primers (P1/P2, P3/P4, P2/P3 and P1/P4). Results indicated that the ICE*Ssu*1112S integrated on the chromosome maintained the ability to form circular DNA and be excised from chromosomes. However, a 428 bp PCR product was obtained by the primer P4/P1 in S. suis P1/7, indicating the absence of an ICE. Furthermore, conjugation experiment revealed that ICE*Ssu*1112S transferred into the recipient strain S. suis P1/7 with a frequency of 5.9 × 10^−6^ transconjugants per donor cell. The positive transconjugants were further determined by detecting the mobilization of the antimicrobial resistance profile and the gene *optrA*, and the location of the ICE in S. suis P1/7 was detected by PCR and sequencing with primers P1/P2 and P3/P4. The antimicrobial susceptibilities of the donor strain 1112S, the transconjugant T-1112S and the recipient strain S. suis P1/7 were shown in Table 1. The transconjugant T-1112S exhibited elevated MICs of nine antibiotics, including linezolid, compared with the recipient strain. The result of PCR using the primer P1/P2 and P3/P4 showed that ICE*Ssu*1112S was integrated into the P1/7 and the integration site was same with the donor strain (Fig. S1).

In summary, we characterized a *lnu*(C)-positive S. suis isolate that contained a novel multiple antibiotic resistant ICE of ICE*Sa*2603 family carrying the linezolid resistance gene *optrA*, named ICE*Ssu*1112S, which also contained a novel bacitracin resistance locus. In addition, the ICE*Ssu*1112S was self-transmissible and integrated into the genome of S. suis p1/7. Our findings suggested that ICE*Ssu*1112S may serve as an important reservoir for antibiotic resistance genes to accelerate the transfer of resistance genes in *Streptococci.* Therefore, more studies should be needed to access the real contribution of these MGEs to the horizontal transfer of resistance genes, especially for the linezolid resistance gene *optrA* and bacitracin resistance locus.

## MATERIALS AND METHODS

### Bacterial isolates, PCR experiment, and antimicrobial susceptibility testing.

The Streptococcus suis strain 1112S was isolated from a diseased pig in a feedlot from Henan, China, 2019 (34). Linezolid resistance was assessed using broth microdilution. The presence of *optrA* gene was detected by PCR using the primers listed in Table S1. Antimicrobial susceptibility testing was performed using the broth microdilution method according to CLSI guidelines (35), and S. pneumoniae ATCC 49619 and Escherichia coli ATCC 25922 were used as quality control strains.

### Serotyping and multilocus sequence typing (MLST).

Serovar of the strain 1112S was determined using the primers previously described (36). MLST was performed to identify the sequence type of 1112S (37). In brief, seven housekeeping genes (*aroA*, *cpn60*, *dpr*, *gki*, *mutS*, *recA* and *thrA*) were amplified and sequenced. The allelic number and sequence type of 1112S was then determined by comparing the internal fragment sequence of their respective housekeeping genes with the available date from the S. suis MLST database (https://pubmlst.org/ssuis/info/primers.shtml).

### Whole-genome sequencing (WGS) and analysis.

The genomic DNA of strain 1112S was extracted using the TIANamp Bacteria DNA kit (TIANGEN, Beijing, China) as recommended by the manufacturer and sequenced using Illumina Nextseq 449 and the Oxford Nanopore Technologies (ONT) MinION platforms. Sequencing reads, including short-read and long-read data were assembled using Unicycler 0.4.4 with the hybrid strategy (38). The whole-genome sequence was initially annotated using the RAST server (http://rast.nmpdr.org) and corrected by blast manually. Mobile genetic elements were identified using Mobile Element Finder (https://cge.cbs.dtu.dk/services/MobileElementFinder/). A comparative alignment was conducted using Easyfig software (39).

### Cloning of the bacitracin locus.

To verify the function of the putative bacitracin locus, the intact bacitracin locus was amplified by PCR using primers BAC-F (5′-CGCGGATCCCGGAGCTAGACATCAAACTGACTC-3′) and BAC-R (5′-CTAGTCTAGACAAGGAATGCTCTTTCGACTAGC-3′) to introduce two restriction sites (BamHI and XbaI). The resulting PCR product was digested, purified, and ligated to the same restriction sites of the cloning site of E. coli–E. faecalis shuttle vector pAM401. The ligation product was first transformed into E. coli DH5α and the positive clones were confirmed by PCR. The recombinant plasmid was extracted from DH5α using plasmid midi kit (Qiagen, Hilden, Germany) and subsequently introduced into E. faecalis JH2-2 by electroporation (MicroPulser; Bio-Rad, USA).

### Conjugation experiments.

The integrated form and extrachromosomal circular form of the ICE*Ssu*1112S element was detected by combination primers P1–P4 (Table S2) targeting the two terminal sequences of ICE (23). Conjugation assay was performed using S. suis 1112S (linezolid-resistant but rifampin-susceptible) as the donor and S. suis serotype 2 strain P1/7 (ST1) (linezolid-susceptible but rifampicin-resistant) as the recipient, by filter mating experiment according to a previously described method (23). Selection of transconjugants were performed on BHI plates containing 200 mg/liter rifampicin and 4 mg/liter linezolid. The mating assay was conducted at least three times and the frequency of transfer was calculated as the mean number of transconjugants per donor cell. All the transconjugants were confirmed with PCR using the primer *optrA* gene and P2/P3, and susceptibility testing.

### Data availability.

The complete sequences of the chromosome and ICE*Ssu*1112S in S. suis 1112S have been submitted to GenBank under the accession numbers CP071697 and MW790610, respectively.
