# Supplementary material for: Characterization of a Novel Linezolid Resistance Gene optrA and Bacitracin Resistance Locus-Carrying Multiple Antibiotic Resistant Integrative and Conjugative Element ICESsu1112S in Streptococccus Suis
Source: Microbiol Spectr. 2022 Feb 16;10(1):e01963-21. doi: 10.1128/spectrum.01963-21 (PMC8849049; doi:10.1128/spectrum.01963-21)
Supplement: SUPPLEMENTAL FILE 1 — Supplemental material. Download SPECTRUM01963-21_Supp_1_seq7.pdf, PDF file, 0.4 MB [file spectrum01963-21_supp_1_seq7.pdf]

Table S1. Primers used for PCR to detect resistance genes

primer	Sequence 5'–3'	Annealing temperature(°C)	Amplicon size (bp)
optrA-F	TCCTTCTTAACCTTCTCCTTCTCA	54	794
optrA-R	GCACCAGACCAATACGATACAA		
cfr-F	TGAAGTATAAAGCAGGTTGGAGTCA	64	746
cfr-R	ACCATATAATTGACCGACAAGCAGC		
poxtA-F	GAACGCTTGGAGTATTTCGACTTC	55	778
poxtA-R	CTGGACTGAGAATACCCATC		
cfr(B)-F	ACCAACTTGATGATGCGGTAG	55	1912
cfr(B)-R	CCTGGATTAGCACCATCCCA		
cfr(C)-F	GCAACTGCTTGATGCTGTGTT	57	913
cfr(C)-R	GGAGCCAAATTGCGTTCGG		

Table S2. Primers used for PCR to detect excision of ICE*Ssu*1112S

Primers	Sequence (5'-3')	Reference
Primers for detection of ICE*Ssu*1112S		
virB-F	GGTCGAATGGGTCTTTGTCT	(1)
virB-R	GCTTGGTGTGTTGTGGAATC	(1)
int-F	CAAAGGTGTTTATTGTGATA	(2)
int-R	TGGTCTTTACGATACCAAT	(2)
Relaxase-F	GAGGACGCCAACTCAGCAAACG	This study
Relaxase-R	GTCAGTTTGATGTCTAGCTCCGC	This study
Detecting integration/excision form of ICE*Ssu*1112S		
P1	GCGGTCGATAGGAACAACC	(3)
P2	GCAGCAAGTAGGAGTGCCTGTT	This study
P3	AACAGCCAAGCAGTGCCATGA	This study
P4	AAAGTTGGCGTTATCAAAG	(3)

1. Palmieri C, Magi G, Mingoia M, Bagnarelli P, Ripa S, Varaldo PE, Facinelli B. 2012. Characterization of a *Streptococcus suis tet*(O/W/32/O)-carrying element transferable to major streptococcal pathogens. Antimicrob Agents Chemother 56:4697-702. https://doi.org/10.1128/AAC.00629-12.
2. Huang J, Liang Y, Guo D, Shang K, Ge L, Kashif J, Wang L. 2016. Comparative genomic analysis of the ICE*Sa2603* family ICEs and spread of *erm*(B)- and *tet*(O)-carrying transferable 89K-subtype ICEs in swine and bovine isolates in China. Front Microbiol 7:55. https://doi.org/10.3389/fmicb.2016.00055.
3. Pan Z, Liu J, Zhang Y, Chen S, Ma J, Dong W, Wu Z, Yao H. 2019. A novel integrative conjugative element mediates transfer of multi-drug resistance between *Streptococcus suis* strains of different serotypes. Vet Microbiol 229:110-116. https://doi.org/10.1016/j.vetmic.2018.11.028.

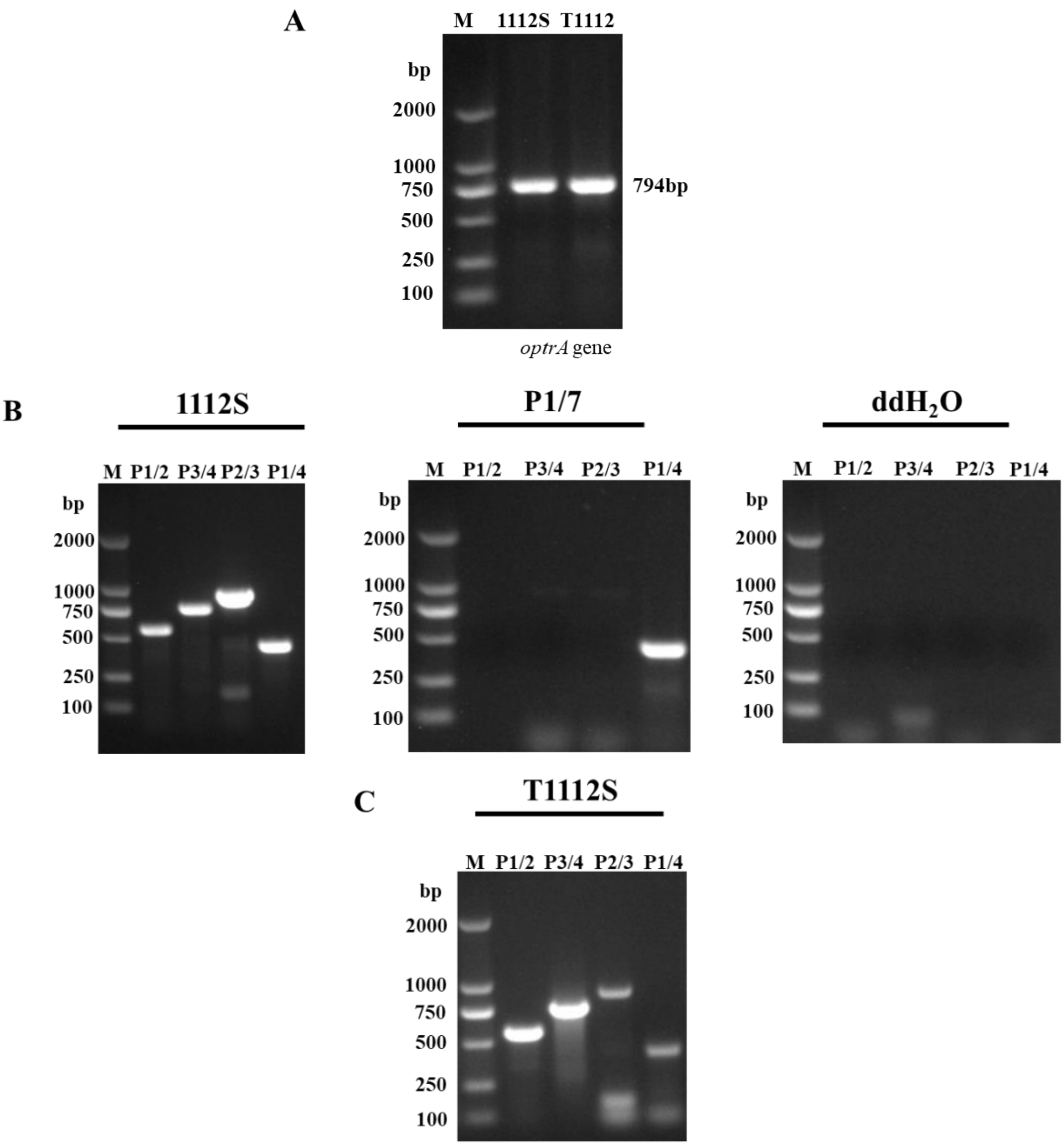

Figure S1. Detection of *optrA* gene and the circular form of ICE*Ssu*1112S in 1112S and transconjugant T1112S, primer pairs are indicated above the lanes.
